# The X-Rays

**Published:** 1912-08-17

**Authors:** Alfred C. Norman

**Affiliations:** House Surgeon at Durham County Eye Infirmary.


### ELECTRICITY IN MODERN MEDICINE.*

### XVI.—The X-Rays.

By ALFRED C. NORMAN, M.D. Edin., House Surgeon at Durham County Eye Infirmary.

In addition to the *x*-ray tubes already described, mention must be made of the so-called "safety" tubes designed for giving localised therapeutic applications to various parts of the body. The glass of which these tubes are made contains a large amount of lead, and is, therefore, practically impervious to *x*-rays, but at one point there is a small round window of soda-glass through which the rays are allowed to emerge. Such tubes are made in various patterns for different purposes. For instance, when constructed with a long narrow prolongation, at the end of which is placed the soda-glass window, they may be used for treating lesions in the mouth, rectum, or vagina; the tubular prolongation being easily passed into the cavity in question. Similar tubes have been used for radiographing the teeth (with the photographic film outside, and the prolongation inside the mouth), but they will not stand sufficiently large currents to give satisfactory results. Therapeutic tubes are sometimes made without an anticathode, and these, of course, give rise to *x*-rays wherever the cathode stream strikes the glass; they have the advantage of being cheap, but can only be used with very small currents, otherwise the glass would be melted. Before the genius of Professor Jackson gave us the focus tube all *x*-ray tubes were made in this way.

To sum up with regard to *x*-ray tubes; for every tube there is a certain "normal" current which will keep that tube in a satisfactory condition. The "normal" current can be determined by watching the milliampere-meter while the tube is working. If, with a certain current in the primary circuit, the milliamperage in the secondary circuit steadily increase, we know that the normal current has been exceeded and that this excess is softening the tube—and, of course, vice versa.

It might be asked why, with a constant amperage in the primary circuit, does the milliamperage in the secondary circuit vary with the resistance? It is simply a matter of wattage and the explanation is as follows. The voltage induced in the secondary circuit of a coil depends entirely upon the resistance to be overcome in that circuit; if the resistance be great the voltage will rise to overcome it, and vice versa. Now the number of watts induced in the secondary circuit must correspond approximately with the number flowing through the primary circuit, hence, when we have our wattage in the secondary circuit in the form of increased voltage it must be at the expense of a correspondingly diminished amperage.

To give a specific example. Let us suppose that a current of 1,000 watts flowing through a primary circuit has to generate 500,000 volts in the secondary circuit to overcome the resistance of a certain tube; then the utmost amperage that can be generated in the secondary circuit is two-thousandths of an ampere—*i.e.*, 2 milliamperes. For the secondary current must be approximately 1,000 watts, and we have seen that amperes × volts = watts; hence,

$$\frac{1{,}000 \text{ watts}}{500{,}000 \text{ volts}} = \frac{2}{1{,}000} \text{ ampere.}$$ That is, 2 milliamperes.

Now, if this current of 2 milliamperes happened to be more than the normal current of the tube, the resistance of the latter would steadily become less and the milliamperage would gradually increase, and thus we should have a vicious circle that would quickly render the tube perfectly useless. The remedy is to reduce the current in the primary circuit until the milliamperage in the secondary is as nearly as possible the "normal" current of the tube.

The "normal" current may be regarded as that current which will keep the tube at such a temperature that the gas liberated from some parts is exactly balanced by the gas occluded in other parts of the tube. It depends, therefore, upon the size of the tube, the thickness and design of the anticathode, and upon the amount of use the tube has already had.

A new tube, on account of its low resistance and on account of the large amount of gas dissolved in its metallic parts, should be used with the utmost caution and vigilance until it becomes seasoned.

The current with which the different *x*-ray tubes can be safely used varies between .2 milliamperes and 5 milliamperes, but very few tubes will stand 5 milliamperes for a prolonged exposure. The writer is quite satisfied if he can get well-seasoned 8-inch Gundelach tubes to stand even 3 milliamperes for any length of time. Small tubes with light anticathodes should not be used with more than about .2 to .5 milliamperes at the outside.

Finally, it is easy to do good work when the tube is behaving satisfactorily; but the radiographer who would keep a set of tubes always in good condition and make them do exactly as he wishes, should acquire some knowledge of their physics. This must be the writer's excuse for introducing so many technicalities.

We now come to consider three pieces of apparatus which are essential to the production of consistent and exact results—namely, the milliampere-meter, to measure the amount of current passing through the tube; the oscilloscope, to indicate the presence or absence of reverse current; and the valve tube (or else a spark gap), to suppress any reverse current that may be present. All of these are connected in the secondary circuit in series with each other and with the *x*-ray tube.

### The Milliampere-Meter.

The milliampere-meter must be of the d'Arsonval type, a description of which can be found in any good text-book of physics. For *x*-ray work the meter is always fitted with a condenser to protect the insulation against the high-tension current. Meters of the "hot-wire" type are absolutely useless for this class of work.

* Previous articles appeared on Nov. 11, 25, Dec. 9, 30, Jan. 13, 27, Feb. 17, March 9, 30, April 20, May 4, 25, June 8, July 6, and Aug. 3.

When a d'Arsonval meter is used to measure a continuous current, as, for instance, in galvanic treatment, the needle indicates exactly the amount of current that is flowing through the circuit, but in the case of a current from an induction coil the problem is not so simple. In the latter case the current consists of a series of individual discharges falling to zero and rising to their maximum many thousands of times in a minute. Now the needle of a meter has a certain amount of inertia which does not permit of its jumping about at such a rapid rate, hence it will not indicate the exact current flowing at a given moment of time, but will come to rest at a point that represents a resultant of the value of each discharge and the rate at which these discharges are produced. In other words, with a current of a given intensity passing through the meter the more rapidly the interrupter is run the higher will be the reading of the meter, simply because the more frequently the discharges follow each other the less time, so to speak, will the needle have between each discharge in which to drop towards zero. Now this is just what we require in *x*-ray work, for we have already seen that the quantity of *x*-rays produced in one second depends not only upon the intensity of each individual discharge, but also upon the number of discharges that take place in that time. Consequently, though the units indicated by the milliampere-meter are not milliamperes in the ordinary sense of the word, they are an absolute indication of the amount of *x*-rays that are being given out by a tube at a given moment, and so they furnish information of the utmost value to the radiographer.

Whenever the term "milliampere" or its abbreviation, "ma.," is used in connection with *x*-ray work it must be understood to mean a unit which embraces both the intensity of the individual discharge and the rate at which the discharges are produced.

The extreme value of this unit to the *x*-ray worker lies in the fact that the speed of the interrupter does not complicate his calculations. For instance, with a tube of a certain penetration the time of exposure for a radiograph will depend entirely upon the number of milliamperes passing through the tube, provided, of course, the distance of the photographic plate from the tube be kept constant. A current of 2 ma. for 10 seconds will have exactly the same effect on a plate as one of 1 ma. for 20 seconds or one of ½ ma. for 40 seconds. Thus we see the product of milliamperes by seconds is a radiographic constant, and an exposure of a certain number of milliampere-seconds will always produce the same result with a tube of the same degree of penetration working at the same distance from the plate.

There is one fallacy to be guarded against however. If there happen to be reverse current passing through the tube the meter reading will not be an accurate indication of the quantity of *x*-rays the tube is giving out, simply because reverse current tends to displace the needle of the meter towards zero while it has no effect upon the quantity of rays which the direct current may be producing. Thus it happens that the meter indicates less current than is really passing and gives us an entirely false impression of the quantity of rays that are being generated—a condition of affairs that may result in an over-exposed plate or a bad over-dose to a patient.

Obviously, when the quantity of reverse current equals the direct current the needle will come to rest at zero though there may be in reality quite a large amount of current passing through the meter. The writer, when using a very soft tube, has even known the reverse current to be in excess of the direct, and then the meter indicated a small current in the opposite direction.

The lesson to be learnt from all this is that the milliampere-meter should not be relied upon unless we see by the oscilloscope tube that there is absolutely no reverse current in the circuit. With this precaution it is the most valuable accessory in our armamentarium.

A meter reading in 10ths up to 5 ma., and provided with shunts so that it may be used for currents up to 50 and 500 ma. respectively, costs about £3 10s. Although at present we can seldom use more than 5 ma. for *x*-ray work, it is advisable to obtain a meter with shunts, because the same instrument can then be used in galvanic treatment. Besides, we do not know what improvements in *x*-ray tubes may be made in the near future.

The vulcanite knob at the top of the meter must be rotated to bring the shunts into operation. When it is turned till the figure 10 appears in the small window it indicates that nine-tenths of the current are shunted past the instrument and consequently that the reading must be multiplied by 10. Similarly, when the figure 100 appears in the window, the reading must be multiplied by 100.

### The Oscilloscope Tube.

We have seen when a tube is exhausted to about the Geissler degree of vacuum that there is sufficient air left in it to give rise to a blue or violet fluorescence along the track of the cathode stream, and we have also seen that a similar cathode beam is produced in an *x*-ray tube which has become very soft.

An oscilloscope tube is simply a narrow tube exhausted to a degree that shows to the best advantage this violet fluorescence. The tube measures about 9 inches in length by about 1½ inch in diameter. Into each end is fused a thin wire electrode and these nearly meet in the middle of the tube. In some patterns they are separated by a mica disc with a small hole in the centre, but this is not essential.

When a current from an induction coil is passed through such a tube the cathode wire inside the tube is surrounded by a violet fluorescence. If there be any reverse current both wires function as cathodes, and there is in consequence a violet fluorescence round each, but it seldom happens that the reverse current is equal in amount to the normal current, hence there is more fluorescence at one wire than the other.

(*To be continued.*)

